# Effectiveness of Polyphenols on Perinatal Brain Damage: A Systematic Review of Preclinical Studies

**DOI:** 10.3390/foods12122278

**Published:** 2023-06-06

**Authors:** Paula Brielle Pontes, Ana Elisa Toscano, Diego Cabral Lacerda, Eulália Rebeca da Silva Araújo, Paulo César Trindade da Costa, Swane Miranda Alves, José Luiz de Brito Alves, Raul Manhães-de-Castro

**Affiliations:** 1 Postgraduate Program of Neuropsychiatry and Behavioral Sciences, Federal University of Pernambuco, Recife 50670-901, Pernambuco, Brazil; 2 Studies in Nutrition and Phenotypic Plasticity Unit, Federal University of Pernambuco, Recife 50670-901, Pernambuco, Brazil; 3 Department of Nursing, CAV, Federal University of Pernambuco, Vitória de Santo Antão 55608-680, Pernambuco, Brazil; 4 Department of Nutrition, Health Sciences Center, Federal University of Paraíba, João Pessoa 58051-900, Paraíba, Brazil; 5 Department of Nutrition, Federal University of Pernambuco, Recife 50670-901, Pernambuco, Brazil

**Keywords:** hypoxia-ischemia, brain, polyphenols, resveratrol, quercetin

## Abstract

Polyphenol supplementation during early life has been associated with a reduction of oxidative stress and neuroinflammation in diseases caused by oxygen deprivation, including cerebral palsy, hydrocephaly, blindness, and deafness. Evidence has shown that perinatal polyphenols supplementation may alleviate brain injury in embryonic, fetal, neonatal, and offspring subjects, highlighting its role in modulating adaptative responses involving phenotypical plasticity. Therefore, it is reasonable to infer that the administration of polyphenols during the early life period may be considered a potential intervention to modulate the inflammatory and oxidative stress that cause impairments in locomotion, cognitive, and behavioral functions throughout life. The beneficial effects of polyphenols are linked with several mechanisms, including epigenetic alterations, involving the AMP-activated protein kinase (AMPK), nuclear factor kappa B (NF-κB), and phosphoinositide 3-kinase (PI3K) pathways. To highlight these new perspectives, the objective of this systematic review was to summarize the understanding emerging from preclinical studies about polyphenol supplementation, its capacity to minimize brain injury caused by hypoxia-ischemia in terms of morphological, inflammatory, and oxidative parameters and its repercussions for motor and behavioral functions.

## 1. Introduction

In early life, the nervous system is much more sensitive to environmental factors. Insults to the central nervous system (CNS) in this period often cause epigenetic adaptations in brain activity and increase the chances of survival [1]. Brain injuries in early life lead to plastic modifications, which are linked to non-progressive disturbances in fetal brain development, intrauterine pathological modifications resulting from toxins or environmental insults, and complications resulting from prematurity. Perinatal hypoxic-ischemic (HI) brain injury brings oxygen deprivation, which results in cerebral insufficiency and cerebrovascular occlusion, and perinatal disorders, e.g., neonatal encephalopathy, leukomalacia, and cerebral palsy [2,3,4,5]. Additionally, diseases resulting from perinatal brain damage are characterized by locomotion, coordination, and sensory perception dysfunctions, which lead to memory, learning and cognition deficits [5,6,7].

These are clinical signs involved with plastic responses resulting from mitochondrial energy impairment; thus, failures in several cascades of the nervous system occur, mainly in antioxidant functions [8], functional immune response [9], and cellular protection [10]. The human cells, including the neurons and glial cells, acquire adaptive responses for energy synthesis under fast anaerobic conditions [1]. However, the anaerobic production of adenosine triphosphate (ATP) cannot supply the energy demand of the CNS for a long time [11,12]. Additionally, anaerobic glycolysis increases the production of free radicals, releasing pro-inflammatory cytokines and resulting in mitochondrial damage and neurodegeneration [1,13,14,15].

Neuroinflammation plays a central role in the genesis of brain injury. Oxygen deprivation increases the production of mitochondrial free radicals, followed by infiltration of leukocytes in the brain parenchyma. This leads to the activation of astrocytes and microglia, causing neuronal death and compromising the development of cerebral white matter [10,11,16]. Furthermore, the release of inflammatory cytokines and T-cell stimulation contribute to neuroinflammation, potentially interfering with central CNS development. Neuronal resistance to hypoxia-ischemia depends, therefore, on the performance of protective mechanisms, including the activation of antioxidant enzymes, reduction of free radical synthesis, and downregulation of pro-inflammatory cascades [17]. These neuroprotective responses are suppressed by microglial activation, leading to neuroinflammation and brain damage, which in turn causes neuronal death, brain atrophy, brain edema and ventricular enlargement [2,18].

In order to comprehend the mechanisms involved with brain injury for perinatal hypoxia-ischemia, experimental HI models have been widely used. This is because they permit analysis of the biochemical and anatomical aspects of the brain tissue. In addition, experimental models can control for the environment, for insult, and for the doses of treatment received, in this case, polyphenols [3,9]. Thus, this type of study brings important pieces of information about the effects of different treatments in brain injury that cannot be observed in clinical studies.

Neonatal hypoxic-ischemic brain injury is an important neurological disorder associated with neonatal death and long-term disability and involves apoptosis, inflammation, and oxidative stress as its primary mechanisms [2,18]. In contrast, some bioactive compounds, such as resveratrol, amentoflavone, mangiferin, and piceatannol may influence these physiological processes. Furthermore, the metabolic pathway stimulated by each compound may vary according to the properties of each [10,16,19,20].

**Figure 1:**
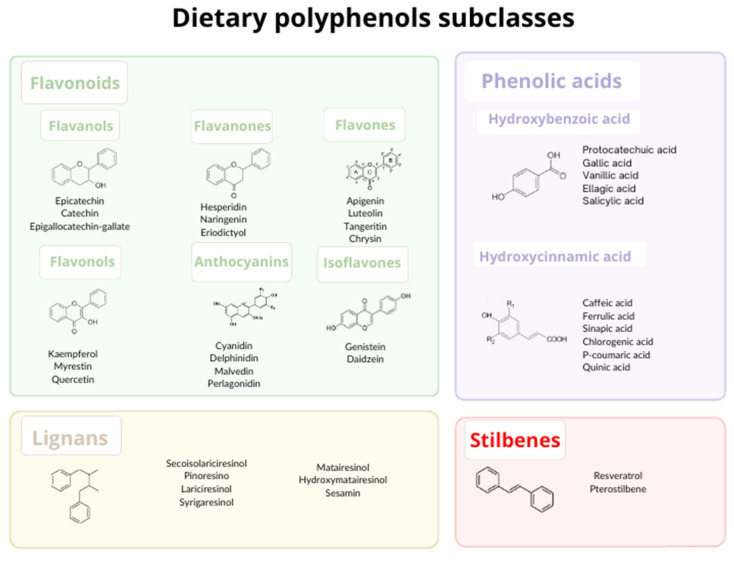
Main classes of polyphenols.

Polyphenols are secondary metabolites found in plants and their products (extracts, tinctures, etc.). Sources of polyphenols include berries, wine, green tea, soybeans, and pomegranate. The basic structure of these polyphenols comprises single or double aromatic rings bonded to one or more hydroxyl groups (-OH) [20,21]. These chemical characteristics permit polyphenols to cross the cell membrane and exert antioxidant and anti-inflammatory action at the mitochondrial and nuclear levels [19,22,23]. Factors such as the type of binding, number of hydroxyl radicals, and aromatic rings differ between classes of polyphenols and give these compounds different bioactive properties (Figure 1) [24,25]. Flavonoids and stilbenes are the most commonly studied, followed by phenolic acids and then lignans [26].

The physiological effects of polyphenols have been widely researched, due to their anti-inflammatory and antioxidant potential against a number of chronic diseases, including neurological disorders [3,8,23,25,26]. Some of the important health advantages of different types of polyphenols include their antimutagenic and anticarcinogenic effects, antimicrobial activity, and activation of the enzymes responsible for xenobiotic detoxification and antioxidant systems [8,11,27,28,29].

Studies have shown the neuroprotective activity of different types of polyphenols in CNS pathologies, including various models of neuroinflammation [11,30,31]. As neonatal hypoxic-ischemic brain injury is a complicated lesion, exploration of the mechanistic effects of different polyphenols as well as the relationship between apoptosis, inflammation, and oxidation is relevant. Several animal studies have indicated that different methods of treatment with polyphenols during pregnancy [32] and lactation [33,34,35] are able to improve locomotor activity, cognitive function, and anxiety behavior in offspring subjected to brain injury. The main neural mechanisms underlying these findings were seen to be the following: reduction of oxidative stress and neuroinflammation in the hippocampus [22,34,36,37], and reduced neuroinflammation with increased remyelination of motor neurons [1,10,26]. The mechanisms by which these polyphenols act on neonatal brain injury, however, have not yet been fully described in the literature [1,10,11].

Considering the pathogenesis of perinatal brain damage, the scientific community has been investigating the use of antioxidant compounds, such as polyphenols, on brain damage in early life [10,11]. Considering the antioxidant and anti-inflammatory properties of polyphenols, we postulate that their effects contribute to a reduction of brain injury caused by HI, although their properties and histopathological mechanisms still require further description. Thus, this systematic review aimed to analyze the effects of polyphenol administration, in experimental models of hypoxia-ischemia in rodents, on brain structures, with particular emphasis on morphological, inflammatory, oxidative, cognition, and behavioral parameters.

## 2. Methods

This systematic literature review was undertaken as per the predefined protocol of PRISMA, or Preferred Reporting Items for Systematic Reviews and Meta-Analyses.

### 2.1. Search Strategy

The literature search was carried out between November 2022 and January 2023, in the electronic databases Medline/PubMed (National Library of Medicine/Medical Literature Analysis and Retrieval System Online), SCOPUS, EMBASE, and Web of Science. The search was based on experimental studies that evaluated the effects of polyphenol treatment on morphological, inflammatory, cognitive, and behavioral function in rats or mice subjected to brain damage in the perinatal phase. There was no restriction on language or year of publication. The standard terms, search strategy, and endpoints are described in Table 1.

### 2.2. Inclusion Criteria

To select the articles present in this systematic review, the following inclusion criteria were established: experimental models that used rodents, induction of perinatal brain damage induced by oxygen deprivation, experimental articles that used a control group in their study design, studies that evaluated the administration of polyphenols (alone or combined) in rodents exposed to hypoxic-ischemic insult, and studies that investigated oxidative stress, biomarkers of neuroinflammation, cognition, and behavior (Table 2).

### 2.3. Exclusion Criteria

Articles were excluded that did not describe in detail the method of inducing brain damage, the source of the polyphenol, the type of polyphenol, the route of administration used, the duration of treatment and the time that supplementation was offered (Table 2).

### 2.4. Characteristics of Studies

The analysis of characteristic studies considered the animal species, the perinatal brain damage induction protocol, as well as the brain damage model. The polyphenol, source, route of administration used, and duration of treatment were also considered (Table 3, Table 4 and Table 5). Primary outcomes were biochemical analysis, antioxidant, and anti-inflammatory parameters, investigation of molecular mechanisms, and morphological parameters of brain damage (Table 6). Secondary outcomes were behavioral assessment, which comprised reflex, motor coordination, sensory, cognitive, and anxiety analysis; and measurement of body weight (Table 6).

### 2.5. Assessment of Risk of Bias

The assessment of the risk of bias was carried out based on the SYRCLE tool, the risk of bias tool for animal intervention studies, which analyses the risk of bias with the following parameters: random sequence generation; baseline characteristics; allocation concealment; blinding of participants; blinding of outcome assessment; random outcome assessment; incomplete outcome data, selective reporting; and other biases, including the inappropriate influence of funders and the presence of unit-of-analysis errors. When the included articles showed a low risk of bias, they were categorized with the symbol “+” (green), and when articles presented a high risk of bias, they were categorized with the symbol “-” (red) (Table 7). The analysis of the risk of bias was carried out by two independent reviewers (BRIELE and LACERDA). When discrepancies occurred between the reviewers, a third reviewer (MANHÃES-DE-CASTRO), who has more expertise in the addressed issues, participated in the judgment of items.

## 3. Results

### 3.1. Selection of Studies

**Figure 2:**
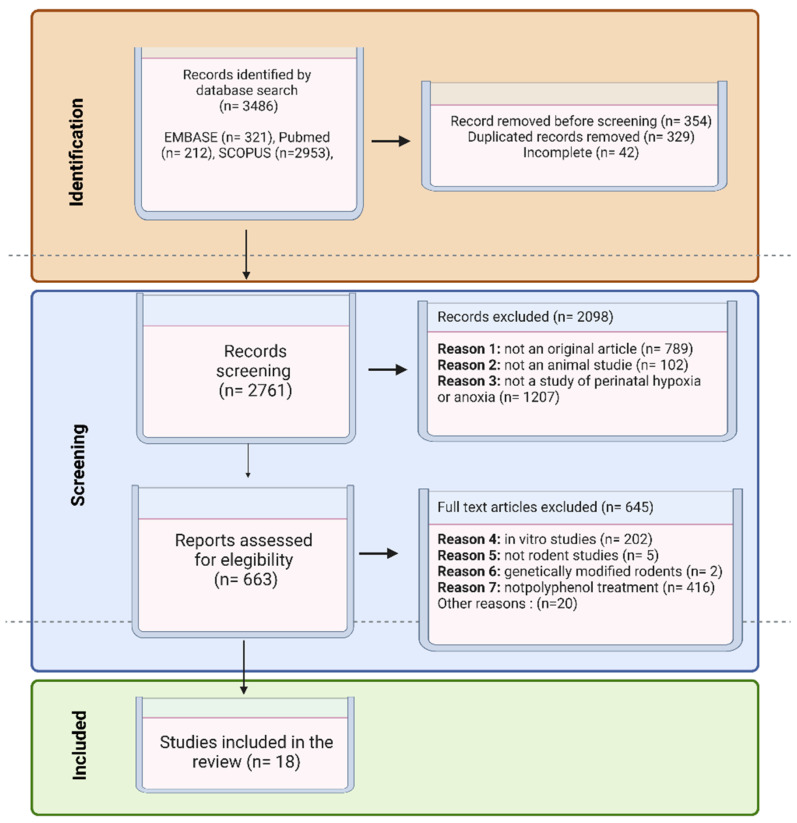
Flow diagram of the article selection process.

An initial database search resulted in 3486 articles. The number of articles identified in each database was as follows: 212 articles in Medline/Pubmed, 2953 articles in Scopus, and 321 articles in EMBASE. After the analysis of the title and abstract, 2098 articles were excluded because they did not meet the inclusion criteria. Finally, after evaluating the full text of the 663 remaining articles, 645 articles were excluded due to disagreement with the eligibility criteria. Therefore, a total of 18 articles were included in this review (Figure 2).

### 3.2. Characteristics of the Studies

The study characteristics included in this systematic review are summarized in Table 3, Table 4 and Table 5 according to the type of animal used (Wistar rats, Sprague Dawley rats, or mice). The animals used in the study were rodents, six Wistar rats, eight Sprague Dawley rats, and four mice. The polyphenols applied in studies to treat perinatal brain damage originated from different sources. Two studies used the analog of resveratrol (trans-resveratrol) [17,33], and two other studies used resveratrol combined with another polyphenol; one with pterostilbene and trans-ε-viniferin [39], and another with stilbenoid, trans-resveratrol, and trans-piceatannol [12]. Eleven articles used isolated resveratrol and, of these, three offered the polyphenol by concentrated pomegranate juice [42,43,44]. A green tea containing polyphenol epigallocatechin gallate as the main active compound was also used in one study [40], while another article used polyphenol amentoflavone [41]. According to the perinatal brain damage model, most of the included studies replicated hypoxia-ischemia induced by unilateral carotid ligation at P7. Two of the studies were an exception to this: one evaluated a childhood hydrocephalus model [40], and the other, brain damage triggered by asphyxia [17]. A wide heterogeneity was observed regarding the route of administration and dose of supplemented polyphenols. Twelve articles used the intraperitoneal route, in doses of 2 μg, 200 μg, 10 mg, 20 mg, 30 mg, 90 mg, and 100 mg/kg from different polyphenol sources. The oral route was chosen in six studies, with varied dosages, of 8, 16, and 32 µmol/day, according to the type of polyphenol administered. In addition, doses corresponding to 0.15, 0.30, and 50 mg/kg were also tested. The administration of polyphenols by oral gavage method, found in one article, corresponded to dosages of 10 or 40 mg/kg [35]. Finally, one study administered doses of 50, 100, or 200 mg/kg through the intragastric route [29]. Treatment duration was also heterogeneous, ranging between 1, 7, 9, 14, and 15 days. In some studies, doses were administered during pregnancy. After birth, administration varied between days and weeks, occurring before or after injury. Moreover, some studies tested the therapeutic potential of polyphenols offered during pregnancy and at the beginning of breastfeeding.

### 3.3. Main Findings

#### 3.3.1. Primary Outcomes

##### Resveratrol

The main outcomes are summarized in Table 6. Resveratrol was the polyphenol most commonly used as the main bioactive compound in the studies. One study observed the ability of post-treatment with resveratrol to protect gray and white substances from brain damage induced by neonatal hypoxia-ischemia [35]. In addition, Pan et al. [35] verified that resveratrol administration after insult reduced the production of inflammatory markers, such as Bax, bcl 2, and caspase-3, leading to an anti-apoptotic effect. Similarly, Le et al. [34] showed that post-treatment with resveratrol led to an anti-inflammatory response in mice exposed to the hypoxia-ischemia model. They found that treatment with polyphenol promoted sirt 1 activation, which in turn inhibited HMGB1, TLR4, Myd88, and NF-KB signaling. Furthermore, another article showed that post-treatment with resveratrol decreased brain damage, downregulated the expression of BAX, and prevented apoptotic death of PC12 cells via miR96 in rodents subjected to hypoxia-ischemia [22]. Another study showed a neuroprotective effect when resveratrol was applied after the hypoxia-ischemia insult [37]. According to this study, a dose of 10 mg of resveratrol per kg and 40 mg/kg was effective to decrease the volume of brain injury in the cortex, hippocampus, and striatum, as well as to enhance hippocampal neurogenesis. Furthermore, the use of resveratrol for 14 consecutive days prevented the fragmentation of mitochondria in the brain region of the resveratrol-treated mice [37].

In contrast, Arteaga et al. [36] found that resveratrol only had a neuroprotective effect when the polyphenol was applied before the hypoxic-ischemic insult. According to this study, pre-treatment with resveratrol reduced the volume of infarct, preserved myelination, and minimized the reactive response caused by brain damage. This is explained by the hypothesis that resveratrol contributes to the maintenance of the integrity of the mitochondrial system and reduces reactive oxygen species [36]. Similarly, Gao et al. [42] have demonstrated that pre-treatment with resveratrol in a dosage of 20 or 40 mg/kg for 7 consecutive days reduced cerebral edema (65.24%), infarct volume (14.30%), lipid peroxidation products and inflammatory markers; increased the Nissl bodies; positively regulated an HO-1 and Nrf-2; and restored the antioxidant state. In addition, according to Revuelta et al. [31], a single application of 20 mg/kg resveratrol before hypoxic-ischemic insult increased brain weight, decreased reactive astrogliosis, and recovered membrane integrity. Dumont et al. [38] used resveratrol to investigate the efficacy of pre- or post-treatment with polyphenol, when applied during the perinatal period. In both situations, resveratrol reduced brain edema and lesion volumes in the cortex, hippocampus, and striatum. Using the resveratrol analog, trans-resveratrol, a study found that the offspring of mothers who received supplementation during pregnancy demonstrated reduced neuroinflammation related to asphyxia induced at P6 [17]. There was also a decrease in the expression of IL-1β, TNF-α-, S-100B, miR132, and miR15a levels, as well as an increase in miR124 and miR123 expression in the hippocampal region [17].

Two studies used pomegranate juice as a source of polyphenols with resveratrol as the main bioactive compound [43,44]. Based on this, Loren et al. [43] verified, through experiments in mice, the efficacy of maternal supplementation with pomegranate juice in neonatal brain protection against hypoxia-ischemia. As a result, there was a decrease in brain tissue loss (60%) and a reduction in caspase-3 activation in the hippocampus (84%) and cortex (64%). Using resveratrol in mice, through pomegranate juice, West et al. [44] also noticed a reduction in the activation of caspase-3; however, they noted that this only happened when the resveratrol was administered before the injury; when administered after 3 h of the insult, it did not show any effect. In addition, they reported that resveratrol decreased calpain activation and protected against brain tissue loss as measured 7 days after injury.

##### Resveratrol Combined with Other Polyphenols

The use of resveratrol, along with viniferin and pterostilbene, has been shown to be efficient at decreasing brain lesions induced by hypoxia-ischemia, in the hippocampus, striatum, and cortex. With regard to combined treatment, Dumont et al. [39], investigated the administration of stilbenoids-trans-resveratrol and trans-piceatannol in the offspring of rats, from mothers who had received alcohol during pregnancy, and who were induced into hypoxia-ischemia. Moderate maternal alcoholism did not increase areas of injury but caused motor deficiencies. Concerning these deficiencies, the trans-resveratrol did not present a reversive effect; however, the trans-piceatannol was able to reverse all sensorineural and cognitive functions. The effective dose of trans-piceatannol in this study was equivalent to one passion fruit per day [12].

##### Other Polyphenols

Using only piceatannol, a flavonoid found in grapes, berries, and passion fruit, a study revealed that maternal supplementation during pregnancy and lactation led to the protection of neonatal brain damage and the reversal of sensory deficits, both motor and cognitive, induced by hypoxia-ischemia in P7 [32]. Amentoflavone, a bioflavonoid, was also administered as a treatment for hypoxia-ischemia induced in young Sprague Dawley rats. In this study, a dose of 30 mg/kg reduced hypoxia-ischemia-induced brain tissue loss. The polyphenol was also efficient at decreasing cell death, lipopolysaccharides, and nitric oxide, as well as blocking caspase-3 activation and proteolytic cleavage of post-lesion substrates [41]. Additionally, mangiferin has demonstrated a neuroprotective effect in rats exposed to hypoxia-ischemia at P10. The P13K/Akt pathway, deregulated after the insult, was activated by the polyphenol, and there was also a decrease in the volume of cerebral infarct and apoptosis, along with a reduction in the levels of expression of caspase-3, Bcl-Xl, and Bcl-2 [29]. One article used green tea containing epigallocatechin gallate as the main bioactive compound, to analyze oxidative stress in rats induced by hydrocephalus [40]. A single daily dose of 50 mg/kg of polyphenol for a period of 15 days reduced the levels of malondialdehyde of the periventricular white substance [40].

#### 3.3.2. Secondary Outcomes

Regarding secondary outcomes which include behavioral parameters, different polyphenol sources were able to alleviate learning, memory, and motor deficits. A single application of resveratrol immediately after a hypoxic-ischemic insult improved motor coordination and cognitive performance [33]. A similar result has been found by Le et al. [34], who showed an improvement in motor parameters in rodents treated with resveratrol offered in a single application after hypoxic-ischemic brain damage. Post-treatment with resveratrol offered over the course of 14 days, was also found to improve cognition function, as well as to decrease antidepressant behavior and anxiolytic effects [37]. According to Arteaga et al. [36], a single application of resveratrol, 10 min before hypoxia-ischemia induction, decreased anxiety and neophobia and improved spatial and non-spatial working memory. In addition, isolated or combined treatment with resveratrol throughout the gestation and lactation periods was able to improve sensorimotor and cognition parameters [12,32,39]. Treatment with Piceatannol also improved locomotion and memory parameters [32].

### 3.4. Analysis of Risk of Bias

The analysis of the risk of bias in the articles included was performed according to the adapted Systematic Review Centre for Laboratory Animal Experimentation (SYRCLE) tool; this consists of nine issues related to four types of bias: selection bias, performance bias, detection bias, and other biases. After analyzing the studies, we observed methodological deficits in all included articles (Table 7). All of the 18 included studies referred to the randomization of the sample; however, they did not report the applied method. Most articles adequately described the conditions of animal care (room temperature, water, and diet). Of all the included studies, only [31,36,43], and [32] performed blinding interventions. However, it is important to highlight that blinding animal interventions are almost unfeasible due to the induction of HI brain injury, which limits the ability to keep animal identities unknown. Additionally, the phenotype of animals submitted to HI models could have informed the evaluators about animal identity, which limits blinding methods. Eight studies [12,32,33,36,38,39,41,43] blinded the evaluation methods, which may have increased the accuracy and reliance of extracted results.

## 4. Discussion

Preclinical studies have demonstrated the potential of polyphenol supplementation during early life towards attenuating neuroinflammation and brain injury in an experimental model of perinatal hypoxia-ischemia. Experimental evidence has revealed that oxygen deprivation can increase pro-inflammatory cytokines and oxidative stress. Conversely, according to the included studies, the different methods of treatment with polyphenols that were offered during the perinatal period were able to alleviate brain damage triggered by HI. Neuroprotection induced by polyphenols can be explained by a number of mechanisms, including decreased neural cell death, reduced apoptosis markers, and increased expression levels of brain SIRT 1, Bcl 2, and SOD 2, mainly in the striatum and hippocampus [30,41]. In addition, therapy with polyphenols upregulated the expression of miR 124 and miR 134 in the cortex, striatum, and hippocampus, indicating greater epigenetic adaptation [45]. Thus, neuroprotective effects induced by polyphenols seem to be more relevant in the striatum, cortex, and hippocampus and reproducible between individual polyphenol or extract treatments. This information will allow the development of studies employing other sources of polyphenols, facilitating analysis of their intervention in specific parts of the neurological pathways involved in anatomic, physiological, and behavioral adaptations in HI animal models. The following sections will present and discuss the underlying mechanisms related to neuroprotection induced by perinatal supplementation with polyphenols.

### 4.1. Neuroprotective Role of Polyphenols

#### 4.1.1. Polyphenols Reduced Morphological Damage

Hypoxia-ischemia models induced morphological damage. According to a number of included studies, periventricular white matter is one of the most vulnerable sites affected by ischemic changes. Hypoxic-ischemic insults, when occurring during the perinatal period, induced significant evidence of infarction, a marked increase in the number of dying cells, characterized by shrunken cells with pyknotic nuclei, and the appearance of swollen and deformed neurons [36,40]. In addition, the studies selected showed that hypoxia-ischemia can lead to brain atrophy and gross enlargement of the cerebral ventricles [1,40]. Conversely, a number of included studies demonstrated that the majority of analyzed polyphenols sources diminished tissue loss and consequently the infarct area [41,43].

However, it is pertinent to highlight that the therapeutic potential of polyphenols depends on the dose and the time of administration. Included articles showed a great heterogeneity of the applied dose of polyphenols, varying from low-middle to high doses of these bioactive compounds; however, it seems that high doses of polyphenols (for instance 20–90 mg/kg of resveratrol) were more effective in attenuating neural tissue injury [37,42,43]. Regarding the time of administration of polyphenols, it seems that administration of polyphenols before the hypoxic-ischemic event is more effective in inducing neuroprotection [36,41,44]. Animals that received resveratrol before hypoxic-ischemic events decreased the appearance of neuropathological signs, reduced infarct volume and alleviated cell damage [36]. In addition, according to included studies, it seems that no adverse effects or reproductive toxicity were observed in animals treated with polyphenols, independent of the dose and the time of treatment [26]. Therefore, we highlight that the supplementation of different sources of polyphenols is well-tolerated, with low toxicity, which justifies the use of these bioactive compounds in many controlled clinical trials [21,26]. Taken together with this data, polyphenols, especially resveratrol, when administered before hypoxic events, could preserve the morphology of the CNS and prevent dysfunction in the affected neurons.

#### 4.1.2. Polyphenols Alleviate the Inflammatory State

One of the main mechanisms that explains robust neuroprotection triggered by polyphenol treatment is related to its anti-inflammation properties. Among tested polyphenols, resveratrol was able to decrease microglial activation. The production of pro-inflammatory factors, including proinflammatory cytokines such as TNF-α, IL-18, IL-1β, and IL-6, in the hippocampus and cerebral cortex, attenuated cerebral deterioration induced by an ischemic event [17,37,42]. Coupled with the modulation of microglial activity, an intraperitoneal single application of resveratrol in a dosage of 20 mg/kg influenced the function of astrocytes, reducing astrogliosis in rodents treated by polyphenols [10,30,31,36]. Excessive astrogliosis impairs neuronal signaling and contributes to the disruption of myelination [33]. Morphological features were also preserved due to the capacity of polyphenols to reduce myelination deficiency in rodents subjected to hypoxia-ischemia models, in subcortical white matter, including the corpus callosum, external capsule, and striatum [33,46]. Oligodendrocytes are expressively responsive to perinatal brain damage induced by hypoxia-ischemia models, leading to white matter injuries [31]. Another important underlying mechanism is that different sources of polyphenols, including resveratrol, amentoflavone and mangiferin supplementation, prevents deficits in morphological parameters due to their potential to decrease hypo-induced caspase-3 activation, which reduces apoptotic index [29,43].

#### 4.1.3. Polyphenols Attenuated Caspase-3-Activity

According to Loren et al. [43], supplementing the mouse maternal diet with pomegranate-derived polyphenols (concentrated juice containing 8, 16, or 32 µmol of polyphenols, per day for 8 days in the uterus and 7 days in the neonatal period), promotes significant neuroprotection against HI injury, even in the low dose group. These findings are associated with a reduction of caspase-3 enzyme activation. This enzyme is an important biomarker of cellular apoptosis. Recently it has been reported to be associated with synaptic and dendritic neurodegenerative processes. Corroborating this, other studies have observed decreased brain injury after perinatal oral supplementation of polyphenols (resveratrol and piceatannol mix, 0.15 mg/kg, per day, during the last week of gestation until p9) [12,32]. The researchers also observed a reduced % of damaged brain area and decreased caspase-3 enzyme activation.

Evidence suggests that bioactive compounds such as resveratrol, mangiferin, and amentoflavone can stimulate the anti-apoptotic pathways [35,37,41]. According to Shin et al. [41], amentoflavone acts by inhibiting caspase-3 and preventing cells from undergoing irreversible actions that culminate in nuclear condensation and DNA fragmentation. Resveratrol may also act by decreasing caspase-3 activity, as well as altering the expression of other apoptosis-related genes, such as Bax and Bcl-2 [11,19,22,35,38]. Mangiferin can activate the PI3K/Akt pathway, because there is the involvement of the mTOR multiprotein complex, leading to cell survival and differentiation and cell metabolism, which is involved in neurocyte nutrition [13,37].

#### 4.1.4. Polyphenols Decreased Oxidative Stress Markers

It has been reported that resveratrol and its analog piceatannol can activate transduction mechanisms enabling improvement of the antioxidant profile in hypoxia-ischemia models [32,36]. Resveratrol can improve mitochondrial function. One of its mechanisms is through the activation of the AMPK signaling pathway, which increases the production of NAD+ and consequently adenosine triphosphate (ATP), which may not be available under pathological conditions such as those presented in this review [28,31,33]. In addition, this bioactive compound also acts on the Nrf2-dependent pathway to regulate the synthesis of endogenous antioxidant enzymes such as glutathione peroxidase (GPx), superoxide dismutase (SOD), and catalase (CAT) [47]. Additionally, the antioxidant effects of piceatannol may be linked to the modulation of the central metabolism, because glycolysis would be increased by the greater uptake of glutamate, also increasing the production of lactate, an energy substrate for neurons, and also of glutathione (GSH), which exerts an antioxidant function [12,32].

#### 4.1.5. Polyphenols and Modulation of Metabolic Pathways

The most discussed metabolic pathways related to the mechanisms of action of polyphenols in hypoxic-ischemic brain injury are those that have anti-apoptotic properties, such as the caspase pathway and the phosphatidylinositol 3-kinase (PI3K)/protein kinase B (Akt) signaling pathway. In addition, polyphenols are also capable of activating other signaling pathways, such as sirtuin 1 (sirt 1), nuclear factor-erythroid 2 related-factor 2 (Nrf 2), and AMP-activated protein kinase (AMPK) [34,48,49,50].

Inflammation is an important contributor to brain damage, and microglia are responsible for provoking early and pronounced inflammatory reactions in the immature brain after a hypoxic-ischemic insult. Resveratrol exhibited anti-inflammatory properties through different molecular mechanisms. This polyphenol was able to decrease nuclear factor kappa B (NF-κB)-dependent transcription, causing the suppression of inflammatory genes [34,35]. Furthermore, resveratrol activates sirt 1 to deacetylate high mobility group box-1 (HMGB1) and thus attenuated its translocation, which may have inhibited the inflammatory response mediated by the toll-like receptor 4 signaling pathway (TLR4) downstream [34,47]. Dumont et al. [38] have also highlighted the participation of sirt 1 in the metabolic pathway triggered by resveratrol supplementation in hypoxia/ischemia models.

#### 4.1.6. Polyphenols and Epigenetics Modulation

In order to comprehend epigenetics mechanisms and neuroplasticity, miRNAs have been studied as important epigenetic factors, because they alter gene expression in the face of oxygen deprivation in early life. Bian et al. [22] have observed a decrease in the expression of miRNA96 after administration of resveratrol (intraperitoneally, 100 mg/kg, 0 h, 8 h, and 18 h after HI brain injury). miRNA 96 increases the expression of genes involved in apoptosis processes and induces caspase-3 activation [22,41]. Thus, the reduction of miRNA 96 expression seems to be the epigenetic mechanism by which polyphenols can reduce caspase-3-mediated apoptosis and consequently the extent of brain damage [41].

With a similar respect to epigenetic modulation through miRNA, oral supplementation of mother rats with trans-resveratrol (50 mg/kg per day, during pregnancy until P7) increased the expression of miRNA 124, the most abundant miRNA in the CNS. miRNA 124 induces neuroprotection and increases anti-inflammatory processes, not only during the differentiation of neurons and the development of microglia but also during the polarization of microglia and macrophages [45]. In addition, increased mRNA-relative expression of brain sirt 1, bcl 2, and SOD 2 were observed. These results mean that the administration of polyphenols (both orally and even in low doses), positively regulates neuroplasticity in early life [45].

Animals subjected to HI at P7 and treated with a single intraperitoneal application of resveratrol on the day of insult showed positive effects on the locomotor activity at doses of 20 mg/kg [36], 90 mg/kg [33], or 100 mg/kg [34]. In all of these studies, the animals performed better in the open-field test. The hippocampus, cortex, and striatum are some of the regions most affected by neonatal oxygen deprivation. Evidence in the literature suggests that a reduction of neurogenesis occurs in this region after a hypoxic-ischemic brain injury [1,51]. Associated with this neuroinflammation, perinatal anoxia may cause inappropriate cell migration, with reduced survival of newly generated neurons [1]. The positive effects of polyphenol administration in these brain regions have already been reported in this review and are probably associated with the amelioration of motor performance.

## 5. Conclusions and Future Perspectives

This review provided information on studies involving the beneficial effects of polyphenols on neurological and behavioral parameters in rodent models of perinatal hypoxic-ischemic brain damage. The main findings of the articles included in the study show that treatment with polyphenols led to (1) reduced area loss of brain tissue (2) decreased microglia activation and astrocyte activation, (3) reduced volume brain damage, (4) improved sensory-motor and cognitive function, and (5) reduced anxiety and depressive behavior. These benefits were associated with some neural responses, including reduced oxidative stress and neuroinflammation in different brain segments, including the cerebral cortex, striatum, corpus callosum, and hippocampus regions (CA 1 and CA 3). Treatment with polyphenols also inhibited caspase-3 activity.

Evidence suggests that the beneficial effects found in adulthood could result from permanent alterations in epigenetic regulation and related phenotypes in rodents treated with polyphenols. Studies have proposed that epigenetic changes could stimulate genes related to the phosphatidylinositol 3-kinase (PI3K)/protein kinase B (Akt) signaling pathway. In addition, the studies reported that polyphenols are also capable of activating other signaling pathways, such as sirtuin1 (sirt 1), nuclear factor-erythroid 2 related-factor 2 (Nrf 2), and AMP-activated protein kinase (AMPK), although further studies are required to understand these mechanisms fully. Histone and DNA methyltransferase alterations establish AMPK as a key player in epigenetic regulation. For instance, oxygen deprivation is often associated with reduced AMPK expression, which may be relieved by supplementation with polyphenols. In addition, polyphenol supplementation could stimulate the AMPK pathway, which attenuates the expression of inflammatory mediator genes through NF-κB suppression. The activation of the AMPK pathway also leads to enhanced expression of the antioxidant systems SOD, glutathione peroxidase, and catalase. Concerning the sources of polyphenols, the included studies suggest that supplementation with resveratrol alone or combined with other polyphenols, especially when administered before HI insult, was more effective in alleviating cerebral and behavioral deficits induced by HI. The heterogeneity of these studies, however, as well as the variation of duration, dose, and co-interventions, limit the understanding of the effectiveness of polyphenols in the management of neonatal hypoxic-ischemic brain injury. However, it is important to mention that since all findings came from preclinical studies, future studies are still needed to investigate the potential of polyphenols in humans suffering from diseases caused by hypoxia-ischemia.

## Figures and Tables

**Table 1:** Standard terms used in the search strategy, and the primary and secondary endpoints.

Search Strategy Component	Terms/Booleans Operators	Primary Endpoints	Secondary Endpoints
Rodent models subjected to perinatal brain injury	(Models, Theoretical) OR (Rodentia) OR (Rats) OR (Mice) AND (Brain Injuries) OR (Hypoxia, Brain) OR (Hypoxia-Ischemia, Brain), (Brain Ischemia)	Biochemical analysis	Body weight measurement
Supplementation with different sources of polyphenols	(Polyphenols)	Antioxidant parameters	Reflex and motor coordination
Perinatal brain damage parameters	(Brain) OR (Brain Diseases) OR (Neuronal Plasticity)	Anti-inflammatory parameters	Sensory evaluation
Antioxidant and anti-inflammatory properties	(Antioxidants) OR (Antioxidant Response Elements) AND (Anti-Inflammatory Agents)	Perinatal brain damage evaluation	Cognitive function
Behavioral assessment	(Behavior) OR (Motor Skills) OR (Motor Skills Disorders) (Cognition Disorders)	Molecular mechanisms investigation	Anxiety behavior

**Table 2:** Eligibility criteria.

Inclusion Criteria	Exclusion Criteria
Participants: - Neonatal rodent model (mice or rats) subjected to perinatal/neonatal brain injury.	Participants: - Neonatal rodent model (mice or rats) subjected to brain injury after the perinatal period.
Exposure: - Rodents supplemented by polyphenols during the early stages of life.	Exposure: - Rodents not supplemented with polyphenols.
Control: - Sham.	Control: - Studies without a control group.
Outcomes: - Brain damage assessment; - Antioxidant and anti-inflammatory properties; - Behavioral skills.	Outcomes: - Long-term parameters of brain damage, antioxidant and anti-inflammatory properties, and behavioral skills.
Study type: - Original data; - Full-test was available.	Study type: - No original data (e.g., reviews, editorial).

**Table 3:** Characteristics of studies using Sprague Dawley rat pups.

Reference	Experimental Groups (*n*)	Perinatal Brain Damage Model	Source of Polyphenol (s)	Main Active Polyphenol (s)	Route and Dose of Administration	Duration of Treatment
Karalis et al., 2011 [33]	Group 1: hypoxic-ischemic rats + resveratrol; Group 2: hypoxic-ischemic rats + sham; Group 3: control group.	Hypoxic-ischemic brain damage induced by unilateral carotid ligation at P7.	Trans-resveratrol (Sigma Aldrich, St. Louis, MO, USA).	Resveratrol.	Intraperitoneally (90 mg/kg).	A single application (immediately after hypoxia).
Isac et al., 2017 [17]	Group 1: control group; Group 2: perinatal asphyxia group; Group 3: perinatal asphyxia + trans-resveratrol.	Pups were exposed to a 90-min asphyxia (9% O_2_, 20% _CO2_ and 71% N_2_) on P6.	Trans-resveratrol.	Trans-resveratrol.	Orally, drinking fluid (50 mg/kg).	The diet offered to mothers starting at P30 (after weaning), during gestation, until postnatal day 7.
Dumont et al., 2019 [32]	Group 1: sham group; Group 2: control group (hypoxic-ischemic); Group 3: hypoxic-ischemic + resveratrol (lactation); Group 4: hypoxic-ischemic + resveratrol (gestation + lactation).	Hypoxic-ischemic brain damage induced by unilateral carotid ligation at P7.	Piceatannol (3,3′,4,5′-trans-tetrahydroxystilbene).	Piceatannol.	Orally, drinking fluid (0.15 mg/kg).	Once daily, during the first week of lactation or during the last week of gestation plus the first week of lactation.
Dumont et al., 2020 [12]	Group 1: control group; Group 2: hypoxic-ischemic + ethanol (4 mg/kg); Group 3: hypoxic-ischemic + ethanol (0.5 g/kg); Group 4: hypoxic-ischemic + trans-resveratrol; Group 5: hypoxic-ischemic + trans-piceatannol.	Hypoxic-ischemic brain damage induced by unilateral carotid ligation at P7.	Stilbenoid polyphenols, trans-resveratrol and trans-piceatannol (Sigma-Aldrich, St. Quentin Fallavier, Itère, France).	Trans-piceatannol; Resveratrol.	Orally, drinking fluid (0.15 mg/kg).	Supplementation starts from the last week of gestation to P9.
Dumont et al., 2021 [38]	Group 1: control group; Group 2: resveratrol group; Group 3: hypoxic-ischemic group; Group 4: hypoxic-ischemic + resveratrol (gestation + lactation); Group 5: hypoxic-ischemic + resveratrol (first week of lactation); Group 6: hypoxic-ischemic + resveratrol (gestation); Group 7: hypoxic-ischemic + resveratrol (second week of lactation).	Hypoxic-ischemic brain damage induced by unilateral carotid ligation at P7.	Resveratrol (Sigma-Aldrich, St. Quentin Fallavier, Itère France).	Resveratrol.	Orally, drinking fluid (0.15 mg/kg).	Last week of gestation + first and second week of breastfeeding.
Roumes et al., 2022 [39]	Group 1: control group; Group 2: hypoxic-ischemic group; Group 3: hypoxic-ischemic + resveratrol group (2 weeks before insult); Group 4: hypoxic-ischemic + cocktail group (2 weeks before insult); Group 5: hypoxic-ischemic + cocktail group (2 weeks after insult).	Hypoxic-ischemic brain damage induced by unilateral carotid ligation at P7.	Resveratrol, Pterostilbene, Viniferin (Exinnov, Saint Jean d’Illac, France).	Pterostilbene; Trans-ε-viniferin; Resveratrol.	Orally, drinking fluid (0.15 mg/kg for resveratrol and Pterostilbene, and 0.30 mg/kg for Viniferin).	For 2 weeks (last week of gestation + first week of breastfeeding.
Etus et al., 2003 [40]	Group 1: control group Group 2: hypoxic-ischemic group Group 3: hypoxic-ischemic + EGCG (between 1 and up to 15 days after insult)	Hypoxic-ischemic brain damage induced by unilateral carotid ligation at P7.	Epigalocathecin galate (EGCG) (Sigma-Aldrich, France).	EGCG	Intraperitoneal (50 mg/kg)	For 15 days, daily, after hipoxic ischemic brain injury.
Shin et al., 2006 [41]	Group 1: control group; Group 2: Hypoxic-ischemic + apigenin (6 h after insult) Group 3: hypoxic-ischemic + luteolin (6 h after insult) Group 4: Hypoxic-ischemic + amentoflavone (6 h after insult)	Hypoxic-ischemic brain damage induced by unilateral carotid ligation at P7.	Amentoflavone (Sigma-Aldrich, France).	Amentoflavone	Intraperitoneal (30 mg/kg)	A single application (6 h after hypoxia)
Revuelta et al., 2016 [31]	Group 1: control group; Group 2: hypoxic-ischemic group; Group 3: hypoxic-ischemic + nicotine hydrogen tartrate group (2 h before insult); Group 4: hypoxic-ischemic + melatonin group (10 min after insult); Group 5: hypoxic-ischemic + resveratrol group (10 min after insult); Group 6: hypoxic-ischemic + docosahexaenoic acid (10 min before insult).	Hypoxic-ischemic brain damage induced by unilateral carotid ligation at P7.	Resveratrol (Sigma-Aldrich, France).	Resveratrol	Intraperitoneal (20 mg/kg)	A single application (10 min after hypoxia)

**Table 4:** Characteristics of studies using Wistar rat pups.

Reference	Experimental Groups (n)	Perinatal Brain Damage Model	Source of Polyphenol (s)	Main Active Polyphenol (s)	Route and Dose of Administration	Duration of Treatment
Arteaga et al., 2015 [36]	Group 1: control group; Group 2: hypoxic-ischemic group; Group 3: hypoxic-ischemic group + resveratrol before insult; Group 4: hypoxic-ischemic group + resveratrol after insult.	Hypoxic-ischemic brain damage induced by unilateral carotid ligation at P7.	Resveratrol (Sigma-Aldrich Co., Ltd., Gillingham, UK).	Resveratrol.	Intraperitoneal (20 mg/kg).	A single application (10 min before or immediately after the hypoxic event).
Pan et al., 2016 [35]	Group 1: control group; Group 2: hypoxic-ischemic group; Group 3: hypoxic-ischemic group + resveratrol.	Hypoxic-ischemic brain damage induced by unilateral carotid ligation at P7.	Resveratrol (Sigma Chemical Co., UK).	Resveratrol.	Intraperitoneal (100 mg/kg).	Three applications (0 h, 8 h and 24 h after hypoxic-ischemic injury).
Bian et al., 2017 [22]	Group 1: control group; Group 2: hypoxic-ischemic group; Group 3: hypoxic-ischemic group + ethanol; Group 4: hypoxic-ischemic group + resveratrol.	Hypoxic-ischemic brain damage induced by unilateral carotid ligation at P7.	Resveratrol 3,5,4′- trihydroxy stilbene (Sigma Chemical Co., UK).	Resveratrol.	Intraperitoneal (100 mg/kg).	Three applications (0 h, 8 h and 18 h after hypoxic-ischemic injury).
Xi et al., 2018 [29]	Group 1: control group; Group 2: hypoxic-ischemic group; Group 3–5: hypoxic-ischemic group + mangiferin (50, 100, or 200 mg/kg); Group 6: hypoxic-ischemic group + isoflurane; Group 7–9: hypoxic-ischemic group + mangiferin (50, 100, or 200 mg/kg) + isoflurane.	Hypoxic- ischemic brain damage induced by unilateral carotid ligation at P10.	mangiferin (Sigma-Aldrich, St. Louis, MO, USA).	mangiferin.	Intragastrically (50, 100, or 200 mg/kg).	P3 to P12 and 1 h prior to insult on the day of ischemic induction.
Gao et al., 2018 [42]	Group 1: control group; Group 2: hypoxic-ischemic group; Group 3: hypoxic-ischemic group + resveratrol (20 mg/kg); Group 4: hypoxic-ischemic group + resveratrol (40 mg/kg).	Hypoxic-ischemic brain damage induced by unilateral carotid ligation at P7.	Resveratrol (Sigma-Aldrich, St. Louis, MO, USA).	Resveratrol.	Intraperitoneal (20 or 40 mg/kg).	Seven consecutive days before hypoxia-ischemia induction.

**Table 5:** Characteristics of studies using mouse pups.

Reference	Experimental Groups (n)	Perinatal Brain Damage Model	Source of Polyphenol (s)	Main Active Polyphenol (s)	Route and Dose of Administration	Duration of Treatment
Loren et al., 2005 [43]	Group 1: HI mice + plain water control group Group 2: HI mice + high dose of Pomegranate juice; Group 3: HI mice + middle dose of Pomegranate juice; Group 4: HI mice + low dose of Pomegranate juice; Group 5: HI mice _+_ sugar water Group 6: HI mice + vitamin C.	HI brain damage induced by unilateral carotid ligation at P7.	Pomegranate juice concentrate (Wonderful variety of pomegranates) (POM Wonderful, Los Angeles, CA, USA).	Resveratrol.	Orally, drinking fluid (high dose: 32 µmol per day; middle dose: 16 µmol per day; low dose: 8 µmol per day).	Fifteen days (seven days in utero, eight days during neonatal period).
West et al., 2007 [44]	Group 1: HI mice group + resveratrol 24 h before insult; Group 2: HI mice group + vehicle 24 h before insult; Group 3: HI mice group + resveratrol 10 min before insult; Group 4: HI mice group + vehicle 10 min before insult; Group 5: HI mice group + resveratrol 3 h after insult; Group 6: HI mice group + vehicle 3 h after the insult.	Hypoxic-ischemic brain damage induced by unilateral carotid ligation at P7.	Polyphenol-rich pomegranate juice and resveratrol.	Resveratrol.	Intraperitoneal (high dose: 20 mg/kg; middle dose: 200 μg/kg; low dose: 2 μg/kg).	A single application (24 h pre-injury, 10 min pre-injury or 3 h post-injury).
Li et al., 2020 [37]	Group 1: hypoxic-ischemic group; Group 2: hypoxic-ischemic + resveratrol (10 mg/kg); Group 3: hypoxic-ischemic + resveratrol (40 mg/kg).	Hypoxic- ischemic brain damage induced by unilateral electrocoagulation at 8 watts of the right carotid artery at P7.	Resveratrol (Sigma-Aldrich, Los Angeles, CA, USA).	Resveratrol.	Oral gavage (10 or 40 mg/kg).	Once, daily, for 14 days, starting at P14.
Le et al., 2019 [34]	Group 1: control group; Group 2: hypoxic-ischemic group; Group 3: hypoxic-ischemic + resveratrol group; Group 4: hypoxic-ischemic + resveratrol + EX527.	Hypoxic- ischemic brain damage induced by unilateral carotid ligation at P7.	Resveratrol (MedChemExpress, Monmouth Junction, NJ, USA).	Resveratrol.	Intraperitoneal (100 mg/mL).	A single application (first 2 h after hypoxic-ischemic brain damage).

**Table 6:** Primary and secondary outcomes.

Study	Primary Outcomes	Secondary Outcomes
Etus et al., 2003 [40]	↓ % Area loss of brain tissue loss in hippocampus, cortex, and striatum; ↓ Caspase-3 activity;	The authors did not evaluate secondary endpoints.
Loren et al., 2005 [43]	↓ MDA levels.	No difference in body weight.
Shin et al., 2006 [41]	↓ The concentration of DPPH radical; ↓ % Area loss of brain tissue loss in hippocampus, cortex, and striatum; ↓ Caspase-3 activity; ↓ LPS-induced inflammatory activation of microglia; ↓ In the induction of the inflammatory mediators (iNOS, COX-2, IL-1β, TNF-α-α).	The authors did not evaluate secondary endpoints.
West et al., 2007 [44]	↓ % Area loss of brain tissue loss in hippocampus, cortex, and striatum; ↓ Caspase-3 activity; ↓ Caspase-3 and calpain cleavage.	The authors did not evaluate secondary endpoints.
Karalis et al., 2011 [33]	↓ Degree of damage of the cerebral cortex and the hippocampus; ↑ Diameter of the corpus callosum; ↓ Myelin loss on the corpus callosum.	No difference on righting reflex, improved negative geotaxis and gait reflex; ↑ Motor coordination (evaluated by beam walking test, rope suspension test, rota-rod test; ↑ Cognitive performance (evaluated by Morris water maze test and working memory test).
Revuelta et al., 2016 [31]	↑ Brain weight; ↓ Reactive astrogliosis; ↓ Degree of cerebral MBP loss; Recovered membrane integrity (evaluated by % of positive cells to this cardiolipin binding marker).	↓ Body weight loss.
Arteaga et al., 2015 [36]	↓ % Area loss of brain tissue loss; ↓ Cell loss in the hippocampus and parietal cortex; ↓ Values of neuropathology in the CA 1, CA 2–3, dentate gyrus and parietal cortex; ↓ Reactive astrogliosis; ↓ Degree of cerebral MBP loss in external capsule and striatum; Protected mitochondrial inner membrane integrity, and restored mitochondrial transmembrane potential; ↓ Production of ROS.	↓ Anxiety and neophobia (evaluated by hole-board test); ↑ Spatial working memory (evaluated by spatial alternation task), and ↑ non-spatial working memory (evaluated by novel object recognition test).
Pan et al., 2016 [35]	↓ Brain atrophy; ↓ Tissue loss in the hippocampus and cortex; ↓ Number of dying cells in the cortex; ↓ Expression levels of TNF-α-α, IL-18, IL-1β, IL-6, COX-2; ↓ microglia activation; Anti-apoptotic effect by ↓ a number of TUNEL-positive cells; Anti-apoptotic effect by changes in expression of related genes (↓ Bax, ↑ Bcl-2) in the hippocampus and cortex; ↓ Cleaved caspase-3-positive cells.	The authors did not evaluate secondary endpoints.
Isac et al., 2017 [17]	↓ Expression levels of TNF-α-α, IL-1β, S-100B in the hippocampus; ↑ Hippocampal expression of miR124 and miR134, and ↓ expression of miR132 and miR15a.	The authors did not evaluate secondary endpoints.
Bian et al., 2017 [22]	↑ miR-96 and ↓ Bax expression levels in the hippocampus and cerebral cortex; prevented OGD/R-induced PC12 cell apoptosis via miR-96.	The authors did not evaluate secondary endpoints.
Xi et al., 2018 [29]	↓ Cerebral infarct area; ↓ Neuronal apoptosis as determined by TUNEL assay; Antioxidant activity by ↓ ROS, MDA levels; ↓ Cleaved caspase-3-positive cells; ↑ PI3K/Akt signaling pathway.	The authors did not evaluate secondary endpoints.
Gao et al., 2018 [42]	Cerebral edema, infarct area; Lipid peroxidation products; Inflammatory markers (TNF-α-α, IL-1β, IL-6, NF-jB); Restored antioxidative status by ↑ the activity of GPx, CAT, SOD; Upregulation of HO-1 and Nrf2.	The authors did not evaluate secondary endpoints.
Dumont et al., 2019 [32]	↓ Volume of damaged in cortex, hippocampus, and striatum; ↓ Neuronal death in cortical region and striatum; Improved white matter reorganization by ↑ FA.	Improved in righting reflex test, locomotion test; ↓ Sensorimotor deficits using the mNSS; ↑ The discrimination index by novel object recognition test.
Le et al., 2019 [34]	↓ Brain damage area; ↓ mRNA expression of IL-1β, IL-6, and TNF-α-α; ↓ microglia activation; ↓ TLR4/MyD88/NF-κB signaling; ↓ HMGB1 cytoplasmic localization in microglia; inhibition of HMGB1 nucleoplasmic transfer and extracellular release.	Improved motor function (evaluated by cylinder test, forelimb suspension test, and open field test).
Li et al., 2020 [37]	↓ Brain lesion volume in cortex, hippocampus, and striatum; ↑ Proliferation and neural differentiation of the neural stem/progenitor cells of the hippocampus; ↓ Mitochondrial dynamics injury in the hippocampus.	Improved cognitive function (evaluated by Morris maze test and novel object recognition test); Antidepressant behavior and anxiolytic effects (evaluated by tail suspension test, forced swim test, elevated plus maze, and open field test).
Dumont et al., 2020 [12]	Brain lesion volume in cortex, hippocampus, and striatum.	Improved in righting reflex test and negative geotaxis; ↓ Sensorimotor deficits using the mNSS; ↑ The discrimination index by novel object recognition test.
Dumont et al., 2021 [38]	Brain lesion volume in the cortex, hippocampus, and striatum; ↑ mRNA relative expression of brain sirt 1, bcl 2, SOD 2.	Improved in the righting reflex test; ↓ Sensorimotor deficits using the mNSS; ↑ The discrimination index by novel object recognition test.
Roumes et al., 2022 [39]	↓ Brain edema and lesion volumes in the cortex, hippocampus, and striatum.	Improved in the righting reflex test; ↑ The discrimination index by novel object recognition test.

**Table 7:** Analysis of Risk of bias.

Reference	Random Sequence Generation	Baseline Characteristics	Allocation Concealment	Blinding of Participants and Personnel	Blinding of Outcome Assessment	Random Outcome Assessment	Incomplete Outcome Data	Selective Reporting	Other Bias
Etus et al. (2003) [40]	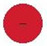	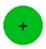	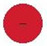	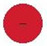	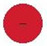	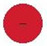	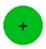	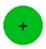	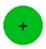
Loren et al. (2005) [43]	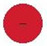	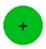	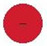	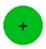	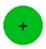	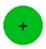	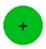	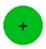	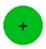
Shin et al. (2006) [41]	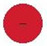	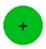	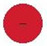	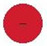	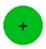	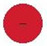	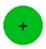	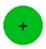	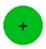
West et al. (2007) [44]	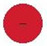	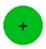	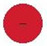	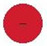	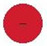	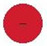	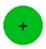	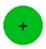	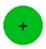
Karalis et al. (2011) [33]	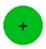	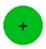	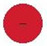	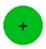	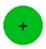	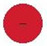	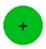	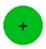	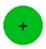
Revuelta et al. (2015) [31]	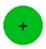	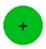	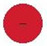	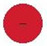	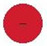	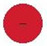	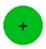	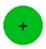	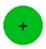
Arteaga et al. (2015) [36]	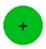	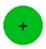	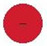	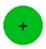	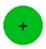	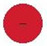	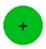	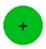	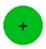
Pan et al. (2016) [35]	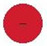	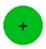	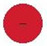	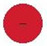	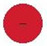	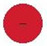	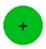	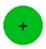	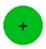
Isac et al. (2017) [17]	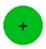	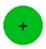	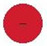	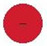	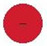	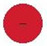	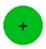	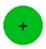	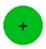
Bian et al. (2017) [22]	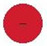	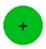	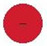	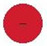	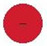	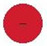	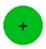	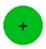	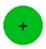
Xi et al. (2018) [29]	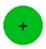	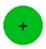	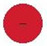	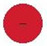	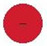	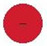	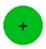	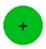	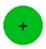
Gao et al. (2018) [42]	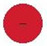	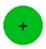	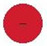	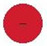	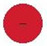	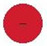	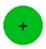	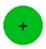	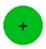
Dumont et al. (2019) [32]	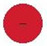	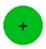	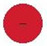	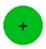	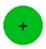	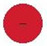	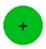	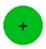	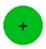
Le et al. (2019) [34]	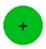	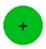	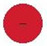	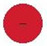	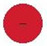	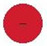	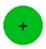	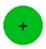	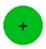
Li et al. (2020) [37]	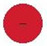	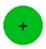	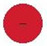	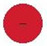	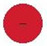	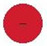	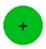	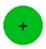	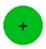
Dumont et al. (2020) [12]	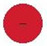	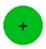	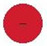	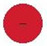	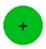	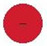	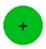	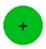	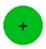
Dumont et al. (2021) [38]	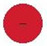	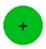	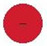	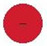	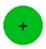	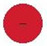	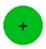	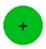	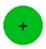
Roumes et al. (2022) [39]	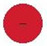	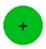	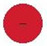	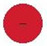	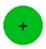	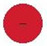	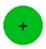	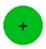	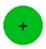

## Data Availability

Data is contained within the article.

## Abbreviations

ATP	Adenosine triphosphate
CAT	catalase
CNS	Central nervous system
COX-2	cyclo-oxygenase-2
DPPH	2,2-diphenyl-1-picrylhydrazy
FA	fractional anisotropy
GPx	glutathione peroxidase
HI	hypoxia-ischemia
HO-1	heme oxygenase 1
IL-1b	interleukin 1b
IL-18	interleukin 18
IL-6	interleukin 6
iNOS	inducible nitric oxide synthase
LPS	lipopolysaccharide
MBP	myelin basic protein
MDA	malondialdehyde
mNSS	modified neurological severity score
NF-jB	nuclear factor kappa B
Nrf2	nuclear factor erythroid 2 related factor 2
PRISMA	Preferred Reporting Items for Systematic Reviews and Meta-Analyses
ROS	oxygen reactive species
S-100B	S-100 beta protein
SOD	superoxide dismutase
TNF-α	tumor necrosis factor alpha
